# Unveiling the protective role of anthocyanin in rice: insights into drought-induced oxidative stress and metabolic regulation

**DOI:** 10.3389/fpls.2024.1397817

**Published:** 2024-05-28

**Authors:** Rahmatullah Jan, Saleem Asif, Sajjad Asaf, Lubna, Zakirullah Khan, Kyung-Min Kim

**Affiliations:** ^1^ Department of Applied Biosciences, Graduate School, Kyungpook National University, Daegu, Republic of Korea; ^2^ Coastal Agriculture Research Institute, Kyungpook National University, Daegu, Republic of Korea; ^3^ Natural and Medical Science Research Center, University of Nizwa, Nizwa, Oman

**Keywords:** anthocyanin, drought stress, melatonin, abscisic acid, antioxidants

## Abstract

This study investigates the impact of anthocyanin treatment on rice plants under drought stress, focusing on phenotypic, molecular, and biochemical responses. Anthocyanin were treated to one month old plants one week before the droughtexposure. Drought stress was imposed by using 10% polyethylene glycol (PEG 6000). Anthocyanin-treated plants exhibited significant enhancements in various traits, including growth parameters and reproductive characteristics, under normal conditions. When subjected to drought stress, these plants displayed resilience, maintaining or improving essential morphological and physiological features compared to non-treated counterparts. Notably, anthocyanin application mitigated drought-induced oxidative stress, as evidenced by reduced levels of reactive oxygen species (ROS) and lipid membrane peroxidation. The study also elucidates the regulatory role of anthocyanins in the expression of flavonoid biosynthetic genes, leading to increased levels of key secondary metabolites. Furthermore, anthocyanin treatment influenced the levels of stress-related signaling molecules, including melatonin, proline, abscisic acid (ABA), and salicylic acid (SA), contributing to enhanced stress tolerance. The enzymatic activity of antioxidants and the expression of drought-responsive genes were modulated by anthocyanins, emphasizing their role in antioxidant defense and stress response. Additionally, anthocyanin treatment positively influenced macronutrient concentrations, particularly calcium ion (Ca^+^), potassium ion (K^+^), and sodium ion (Na^+^), essential for cell wall and membrane stability. The findings collectively highlight the multifaceted protective effects of anthocyanins, positioning them as potential key players in conferring resilience to drought stress in rice plants. The study provides valuable insights into the molecular and physiological mechanisms underlying anthocyanin-mediated enhancement of drought stress tolerance, suggesting promising applications in agricultural practices for sustainable crop production.

## Introduction

Drought is a prominent abiotic stress factor that poses a significant global threat to food security by adversely affecting plant growth and productivity (Seleiman et al., 2021). Its primary association with osmotic stress disrupts cell division and proliferation, hinders normal growth and development, and triggers the overproduction of reactive oxygen species (ROS), collectively leading to diminished yield both quantitatively and qualitatively (Bashir et al., 2021). In recent decades, numerous research studies have proposed that the induction of anthocyanins under drought conditions could serve as a mechanism for osmoregulation and the scavenging of excess ROS (Kaku et al., 1992; Chalker-Scott, 2002). Another formidable abiotic stressor, salinity, imposes simultaneous osmotic and ionic stresses on plants. Perturbed Na^+^ and Cl^−^ homeostasis restricts water and mineral uptake, damages cellular membranes, and diminishes photosynthetic efficiency, consequently reducing crop yield (Isayenkov and Maathuis, 2019). In a manner analogous to their role in responding to drought stress, anthocyanins induced by salt stress have been suggested to alleviate ionic and osmotic damage while scavenging ROS (Eryılmaz, 2006). However, the elucidation of the precise molecular mechanisms underlying the beneficial effects of anthocyanins on plant drought and salt tolerance/resistance has only become possible with the recent application of advanced omics technologies and gene editing tools. These innovative approaches have enabled a more in-depth exploration of the intricate pathways through which anthocyanins contribute to enhancing plant resilience against drought and salt stresses.

Anthocyanins are water-soluble pigments that display hues of blue, purple, or red. They are primarily located in fruits, tubers, and flowers and are produced through the flavonoid metabolic pathway (Khoo et al., 2017). Anthocyanins typically serve multifaceted ecological roles, including the attraction of pollinators and facilitation of seed dispersal. Additionally, they function in photoprotection by shielding against intense light and absorbing ultraviolet radiation. Furthermore, anthocyanins exhibit antioxidative properties by scavenging ROS, contributing to cellular redox homeostasis. Moreover, these pigments play a role in maintaining osmotic balance within plant tissues (Dabravolski and Isayenkov, 2023). Anthocyanins undergo synthesis in the cytosol, initiated by the phenylpropanoid pathway from phenylalanine, forming naringenin chalcone. Enzymatic steps involve early biosynthesis genes such as; *Chalcone synthase* (*CHS*), *Chalcone isomerase* (*CHI*), *Flavanone 3-hydroxylase* (*F3H*), *Flavonol synthase* (*FLS*) and late biosynthesis genes *Dihydroflavonol 4-reductase* (*DFR*), *UDP-glucose: flavonoid 3-O-glucosyltransferase* (*UFGT*), *Anthocyanidin synthase* (*ANS*), *Glutathione S-transferase* (*GST*) for modification (Dabravolski and Isayenkov, 2023). *DFR* plays a crucial role in converting precursor molecules into leucoanthocyanidins, further modified by *ANS* through glycosylation, acylation, and methylation (Saigo et al., 2020). Transport to vacuoles involves *GST* and the MATE (Multidrug and Toxic Compound Extrusion) system. Despite gaps in late modification pathways, transcriptional regulation is driven by *Myeloblastosis Protein* (*MYB*), *Basic Helix-Loop-Helix* (*bHLH*), and *Tryptophan-Aspartic acid 40* (*WD40*) transcription factors, orchestrating the expression of structural genes and downstream regulators (Cui et al., 2021; Khusnutdinov et al., 2021).

Anthocyanins, natural compounds, are produced in response to environmental stresses like low temperatures, salt, drought, nutritional deficiency, pathogen attacks, and heavy metals. They contribute diverse colors (purple, red, blue) to various plant tissues and organs (Kaur et al., 2023). A study on *Brassica napus* (L.) investigated the functional role of anthocyanins in drought defense, focusing on the purple-stem genotype (Chen et al., 2022). The purple-stem genotype exhibited superior antioxidant capability and drought stress tolerance compared to the green-stem genotype. This was evident through heightened expression and activity of antioxidant enzymes, elevated levels of proline and soluble sugar in the purple-stem genotype. These biochemical features enable more effective mitigation of drought-induced ROS and malondialdehyde (MDA) accumulation, demonstrating the protective role of anthocyanins in drought stress response. Additionally, a study on millet (*Panicum miliaceum* L.) investigated the transcriptomic and metabolomic responses to drought stress in the drought-resistant and drought-sensitive varieties (Cao et al., 2022). After 6 hours of drought stress, 97 structural genes associated with anthocyanin biosynthesis were identified. Notably, both varieties exhibited up-regulation of 25 *GST*, 94 *CL*, 8 *F3H*, 4 *PAL*, 2 *CHS*, 2 *CHI*, 1 *DFR*, and 1 *OMT* genes. Additionally, the drought-resistant variety specifically up-regulated 8 *GST*, 3 *F3H*, 2 *OMT*, and 14 *CL* genes. These findings suggest that while both varieties activated anthocyanin biosynthesis metabolically under drought stress, the specific set of up-regulated anthocyanin-metabolism-related genes in drought-resistant plays a crucial role in its drought-resistant phenotype (Cao et al., 2022). Another study shows that, anthocyanin-rich tomato exhibited superior salinity and drought tolerance compared to lower anthocyanin content genotypes, displaying effective regulation of Na^+^ (sodium ion), K^+^(potassium ion), and Ca^2+^ (calcium) levels, higher antioxidant enzyme activity (Superoxide Dismutase (SOD), Peroxidase (POD), and Catalase (CAT)), reduced MDA and ROS production, and less impact on seed germination and root elongation under stress conditions (Napar et al., 2022). Through transcriptomic and metabolomic analysis, it was observed that drought stress induces an increase in anthocyanin content in *A. thaliana* and heightened anthocyanin accumulation was found to mitigate ROS levels in response to drought stress (Nakabayashi et al., 2014). Studies have shown that, elevated anthocyanin levels in transgenic plants have consistently demonstrated heightened resistance to drought, establishing a positive correlation between anthocyanin accumulation and drought tolerance in Arabidopsis, tobacco, and tomato. Overexpression of the snapdragon bHLH transcription factor Delila (Del) in tobacco further validated this correlation, showcasing increased anthocyanin accumulation that enhanced antioxidant capacity, preserved relative water content, and reduced lipid peroxidation in leaves, collectively improving drought stress tolerance (Waseem et al., 2019; Naing and Kim, 2021; Xiang et al., 2021; Li and Ahammed, 2023).

ABA and SA function as key regulators in augmenting abiotic stress tolerance by initiating antioxidant responses, leading to the mitigation of reactive oxygen species such as MDA, hydrogen peroxide (H_2_O_2_), and superoxide ions (O_2_^•−^). Recent research demonstrated that ABA plays a crucial role in governing physiological and biochemical adaptations for drought stress tolerance, with a potential impact on the regulation of plant phenolic compound biosynthesis, particularly anthocyanins that accumulate during drought stress (González-Villagra et al., 2019). Nagira et al. (2006) found that osmotic stress in *Torenia fournieri* plants increased ABA levels before anthocyanin biosynthesis, indicating ABA’s crucial role in modulating anthocyanin induction during drought stress. Meanwhile, González-Villagra et al. (2017) proposed a model suggesting ABA involvement in anthocyanin biosynthesis regulation through microRNA156, enhancing the expression of anthocyanin biosynthesis genes. Despite limited reports connecting changes in endogenous ABA levels to anthocyanin biosynthesis induction, the precise role of ABA in regulating anthocyanin concentrations under drought stress conditions remains unresolved. SA serves as a key plant hormone and elicitor, crucially enhancing plant defense mechanisms against various biotic and abiotic stresses throughout the plant’s life cycle, from seed germination to flowering and fruit ripening (Tiwari et al., 2017; Koo et al., 2020). A study shows that application of SA increased anthocyanins accumulation in grapes until 3 fold (Obinata et al., 2003). Moreover, proline, a significant osmoprotectant, assumes a crucial role in abiotic stress responses. Elevated levels of proline, soluble sugars, and antioxidant enzyme activity were observed in the anthocyanin-rich plant, contributing to a more effective mitigation of the heightened accumulation of ROS and MDA induced by drought stress (Dabravolski and Isayenkov, 2023). Comparable outcomes were observed in lucerne plants overexpressing miRNA156, linking enhanced drought tolerance to elevated levels of ABA, antioxidants, and augmented proline accumulation (Arshad et al., 2017). This suggests that increased proline content in response to drought stress actively contributes to the amelioration of its adverse effects.

The primary objective of our research is to comprehensively investigate the impact of anthocyanins on mitigating drought stress in rice plants. Given the scarcity of published data on this subject, our study aims to provide valuable insights into the influence of anthocyanins on enhancing drought stress tolerance in rice. Additionally, we seek to elucidate the defense mechanisms employed by exogenous anthocyanins to enable rice plants to effectively counteract the adverse effects of drought stress. Through a systematic and thorough assessment, our research will not only contribute to the understanding of the physiological and biochemical changes induced by anthocyanins but also shed light on the intricate pathways through which these compounds bolster the plant’s resilience to drought stress. By elucidating the specific mechanisms involved, we aspire to provide a foundation for the development of sustainable strategies to enhance drought tolerance in rice crops, ultimately contributing to agricultural resilience in the face of changing environmental conditions.

## Materials and methods

### Experimental setup

The current experiment was divided into four treatment groups. The first group consisted of control plants (Cont), second was only anthocyanin-treated (ANs), third was non-treated exposed to drought stress (D), and fourth was anthocyanin-treated exposed to drought stress (ANs+D). Japonica rice (Ilmi cultivar) was selected as a study material and experiment was conducted using three biological replicates. Seeds were sterilized with fungicides and soaked for three days in dark condition at 33°C (Zhao et al., 2023). After successful sprouting the seedling were transferred to pots and kept in dark condition for three days. The experiment was conducted in green house at 28/26°C and 16/8 light/dark condition (Park et al., 2022). The 5 µM anthocyanin was applied to one month old plant, one week before to drought stress exposure. The drought stress was imposed by using 10% polyethylene glycol (PEG 6000) following Jan et al. (2022). Plant length, root length, panicle number, panicle length, number of seeds per panicle and seed weight were measured at final stage of plant growth.

### Analysis of ROS, antioxidant enzyme activity and lipid peroxidation

The hypersensitive response to drought stress was determined by DAB (3,3’-diaminobenzidine) and trypan blue staining. Leaves were collected after one week exposure to drought stress and were subjected to DAB and trypan staining. Leaves of each group were incubated in 1 mg/mL DAB staining for 24 h at 27°C following the method of Chao et al. (2010). DAB stained leaves were then destained by boiling in 95% ethanol for 30 min, cooled down and the brown spots were photographed. For trypan staining, the protocol of Koch and Slusarenko (1990) was followed. In brief, leaves were incubated in trypan blue mixture (2.5 mg/mL trypan blue, 25% w/v lactic acid, 23% phenol, and 25% glycerol) at room temperature for 24 h. Stained leaves were then de-stained with 95% ethanol by shaking overnight at room temperature and photographed after washing with distilled water. Lipid peroxidation was determined by MDA kit and the method are described in detail in (Jan et al., 2021a). CAT, POD, ABTS (2,2’-Azino-bis(3-ethylbenzothiazoline-6-sulfonic acid)) and DPPH (2,2-Diphenyl-1-picrylhydrazyl) were determined by following the method described in detail in (Adhikari et al., 2019; Lubna et al., 2022). H_2_O_2_ contents were quantified by following the method described in (Velikova et al., 2000), while O_2_^•−^ was quantified in rice leaves by following the method described in (Misra and Fridovich, 1972). Plant samples for each type of analysis were collected in three replicates.

### Total RNA extraction and qRT-PCR analysis

To assess the expression levels of specific genes, three leaves were randomly sampled from each treatment group of rice plants following a 24-hour exposure to stress. Total RNA extraction utilized RNeasy Plant Mini Kits (Qiagen, 50), with subsequent cDNA synthesis employing qPCRBIO kits, and qRT-PCR conducted using qPCRBIO SYBR Green kits. Each reaction, comprising 20 µL, initiated with 10 µL SYBR green, 7 µL ddH2O, 1 µL template DNA, and 1 µL of each primer. The thermal cycling conditions included an initial incubation at 95°C for 2 minutes, followed by 35 cycles at 94°C for 10 seconds, 60°C for 10 seconds, and 72°C for 40 seconds. Three independent reactions were performed for each sample on an Eco Real-Time machine (Illumina, Singapore). Actin was employed as the internal reference gene, and relative expression levels were quantified utilizing the 2^-ΔΔ Ct^ method.

### Analysis of ABA and SA

Further we quantified ABA and SA after one week of stress exposure. Leaf samples were collected after one week of drought exposure and freeze-dried in the dryer to separate SA and ABA contents. Dried samples were powdered in liquid nitrogen, and SA and ABA were extracted and quantified by using the Salicylic acid (SA) Elisa Kit from LifeSpan BioSciences and using the Plant Abscisic Acid Elisa Kit from LifeSpan BioSciences (2401 Fourth Avenue, Suite 900, Seattle) (respectively). Both the SA and ABA were quantified by using method mentioned in the user manual.

### Isolation, quantification of anthocyanin, total flavonoids and melatonin

Naringenin, cyanidin, delphinidin, and total flavonoids were extracted using a solvent mixture comprising 70 mL of methanol, water, formic acid, and trifluoroacetic acid in the ratio of 70:27:2:1 (v/v), following the procedure outlined by Jan et al. (2023a). Each sample, with an approximate volume of 2 μl, was combined with 98 μl of 50% acetonitrile (CAN) containing 0.1% formic acid. Subsequent HPLC analysis involved the injection of 1 μl of the prepared sample into the system. The quantification and identification of flavonoids were conducted using an HPLC 1100 Series DAD system (Agilent Technologies, Waldbronn, Germany), equipped with a NUCLEODURH nC18 column (250 mm, 64.66 mm; Pretech Instruments, Sollentuna, Sweden) operating at a temperature of 25°C and a flow rate of 0.8 mL/min. Melatonin was extracted and quantified by using the melatonin extraction kit (Clorometric, cat log No. NBP2-62160) by Novus, USA. The method was followed as described by Jan et al. (2023a). Each of the experiment was repeated three times.

### Na^+^,K^+^, and Ca^+^, and proline quantification

To assess the Na^+^, K^+^, and Ca^+^ concentrations, we harvested fresh shoots from all four treatment groups of rice plants after one week of stress exposure. All the samples were collected in three replicates. Approximately 0.5 g of each sample was finely crushed and homogenized in a solution comprising 7 mL of 65% NHO_3_ and 1 mL of 30% H_2_O_2_. This mixture underwent microwave treatment at 180°C for 20 minutes, followed by a 40-minute cooling period, in accordance with the procedure outlined in (Kang et al., 2021). The resulting solution was then quantified using inductively coupled plasma mass spectrometry (ICP-MS; Optima 7900DV, Perkin-Elmer, Waltham, MA, USA). To quantify proline, 0.5 grams of freeze-dried sample underwent homogenization in 6 N HCl under vacuum conditions for 24 hours, initially at 110°C and subsequently at 80°C. The resulting solid residue was further homogenized in 0.02 N HCl and subjected to filtration through a 0.45 μm filter membrane. Proline analysis was carried out using an atomic amino acid analyzer (L-8900, Hitachi, Japan).

### Chlorophyll and relative water content

To assess the total chlorophyll content, a Soil-Plant Analysis Development (SPAD-502 plus; Konica Minolta Sensing, Seoul, South Korea) meter was employed. Triplicate leaves were randomly selected for each measurement. To quantify the relative water content, ten leaves were systematically sampled from each experimental group following one week of stress exposure. The leaves were promptly weighed upon harvest to determine their fresh weight (FW). Subsequently, these leaves were immersed in distilled water for a period of 4 hours, and their turgid weight (TW) was recorded. Afterward, the leaves underwent a drying process in an oven set at 80°C for 24 hours to ascertain their dry weight (DW). The relative water content (RWC) was computed using the formula:


$$RWC=\frac{FW-DW}{TW-DW}\times 100$$

where FW represents the fresh weight, TW signifies the turgid weight, and DW denotes the dry weight of the leaves. This process was repeated three times.

### Statistical analysis

Statistical analysis was performed on all data using GraphPad Prism software (version 5.01; GraphPad, San Diego, CA, USA). The dataset underwent analysis with a one-way analysis of variance (ANOVA) methodology. Three independent biological replicates were included in the analysis, and means were subjected to comparison through Bonferroni *post hoc* tests. Significance levels were denoted as follows: *P< 0.05, **P< 0.01, and ***P< 0.001.

## Results

### Anthocyanin enhances rice phenotypes in drought stress

**Figure 1:**
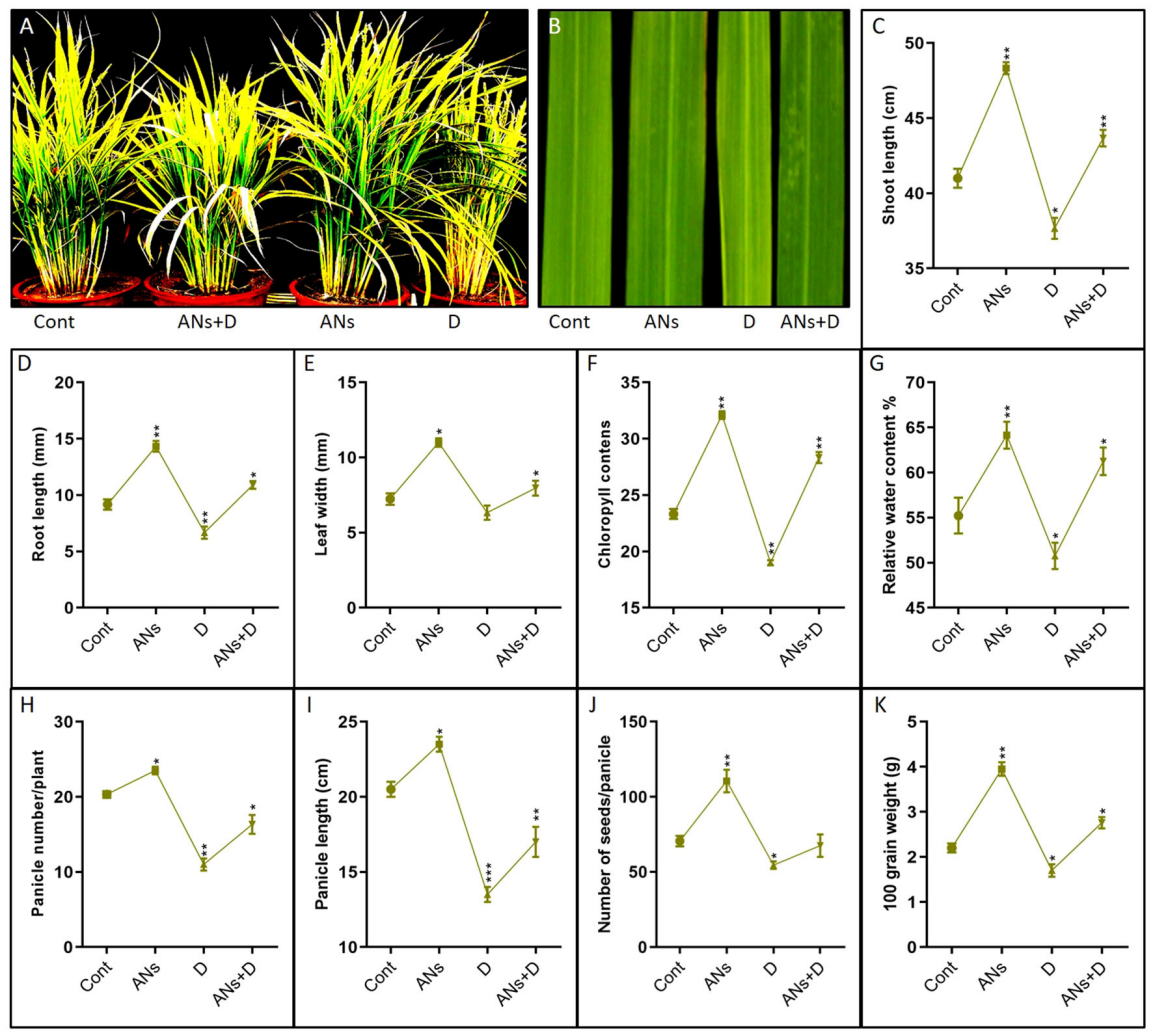
Anthocyanin enhance rice phenotypic traits under drought stress. **(A)** is pictorial presentation of plant length and development, **(B)** is pictorial presentation of leaf width, **(C-K)** Shows shoot length, Root length, Leaf width, Chlorophyll contents, Relative water contents, Panicle number, Panicle length, Number of seeds, and Seed weight of 100 grain respectively. Data in **(C-K)** were from three independent biological replicates ± SD, and means were compared using Bonferroni Post-Hoc test.*P<0.05, **P<0.01, ***P<0.001.

In this study, we assessed the phenotypic response of rice plants subjected to anthocyanin treatment under drought stress, as depicted in **Figure 1**. The data reveals distinctive responses in rice plants treated with anthocyanins under varying conditions of drought stress compared to their non-treated counterparts. In the absence of drought stress, anthocyanin-treated plants demonstrated significant enhancements across multiple traits, including shoot length, root length, leaf width, chlorophyll content, relative water content, panicle number, panicle length, seed number, and seed weight, when compared to control plants (**Figures 1A**–K). Conversely, when non-treated plants were subjected to drought stress, a significant inhibition was observed in all the aforementioned traits. This indicates a potential protective effect of anthocyanin application against the negative impacts of drought on various aspects of plant growth and development. Moreover, under conditions of drought stress, anthocyanin-treated plants displayed noteworthy increases in all assessed traits, except for seed number, in comparison to their non-treated counterparts. This suggests that the exogenous application of anthocyanins may play a role in mitigating the adverse effects of drought stress on rice plants, promoting resilience and maintaining or enhancing essential physiological and morphological characteristics crucial for plant growth and productivity.

### Anthocyanin mitigates drought-induced oxidative stress

**Figure 2:**
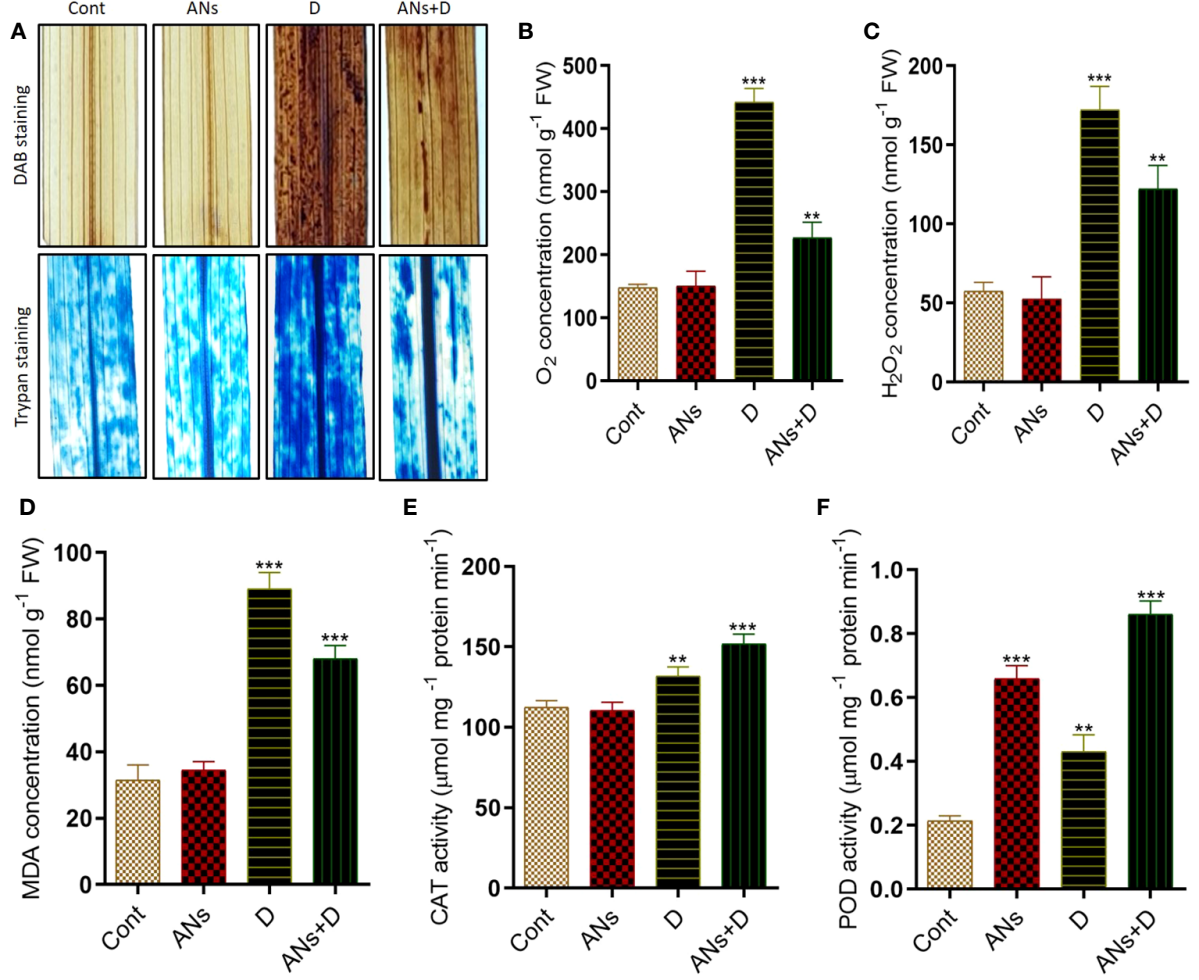
Anthocyanin application reduces oxidative stress induced by drought stress in rice plant. **(A)** shows *In situ* detection of oxidative damage in rice leaves, facilitated by the generation of reactive oxygen species. **(B)** O_2_ concentration, **(C)** H_2_O_2_ concentration and **(D)**. MDA concentration. **(E, F)** shows CAT and POD activity respectively. Trypan and DAB staining were used for O_2_^•−^ and H_2_O_2_ detection. Data were analyzed in three independent biological replicates ( ± standard deviation, SD), and the means were compared using Bonferroni Post-Hoc test. * indicates p< 0.05, ** indicates p< 0.01, and *** indicates p< 0.001.

In our investigation of the alleviation of drought-induced stress through the exogenous application of anthocyanins, we focused on understanding the molecular and biochemical responses in rice plants. To gauge the extent of oxidative stress, we measured the accumulation of key indicators, including H_2_O_2_, O_2_^•−^, MDA, CAT, and POD, under conditions of drought stress. To visually assess oxidative stress induced by drought, we employed DAB (3,3’-diaminobenzidine) and trypan blue staining on the leaves. The results were indicative of pronounced stress in non-treated plants, as evidenced by the appearance of dark brown and dark blue patches in the leaves subjected to drought stress. In contrast, anthocyanin-treated plants displayed a notably reduced occurrence of dark patches under similar drought stress conditions (**Figure 2A**), suggesting a potential protective effect conferred by anthocyanins. Further supporting these staining results, quantification of H_2_O_2_ and O_2_^•−^ in rice leaves revealed higher levels in non-treated plants compared to anthocyanin-treated plants under drought stress (**Figures 2B, C**). Specifically, anthocyanin-treated plants exhibited a substantial 44% reduction in O_2_^•−^ levels and a noteworthy 29% reduction in H_2_O_2_ levels compared to their non-treated counterparts under drought stress. These findings collectively suggest that the exogenous application of anthocyanins contributes to the mitigation of oxidative stress induced by drought, as reflected in both visual and quantitative assessments of key markers associated with ROS accumulation in rice plants.

Lipid membrane peroxidation serves as a crucial biomarker, offering insights into the extent of oxidative damage experienced by plants under stress conditions. This physiological response is traditionally assessed by meticulously quantifying the heightened MDA content. In the context of our investigation, we systematically evaluated MDA levels in response to drought stress, drawing comparisons between conditions with and without anthocyanin treatment. In alignment with observed trends in H_2_O_2_ and O_2_^•−^ accumulation (**Figure 2D**), a discernible increase in MDA content was evident in non-treated plants, surpassing the levels observed in treated plants when exposed to drought stress. Compared to control plants, MDA contents increased by 182% and 115% in non-treated and treated plants, respectively. However, under drought-stress conditions, anthocyanin-treated plants demonstrated a significant reduction of approximately 23% in MDA contents compared to their non-treated counterparts. Concurrently, CAT and POD activities exhibited distinctive patterns of accumulation in both treated and non-treated plants under the duress of drought stress. The application of anthocyanins significantly enhanced CAT activity by 15% and POD activity by 100% in treated plants compared to their non-treated counterparts (**Figures 2E, F**). Interestingly, MDA levels exhibited an antagonistic association with CAT and POD activity under drought stress in both treated and non-treated plants, shedding light on intricate interplays within the plant’s antioxidative defense mechanisms.

### Regulation of endogenous anthocyanins, flavonoids, and biosynthetic gene expression by anthocyanin

**Figure 3:**
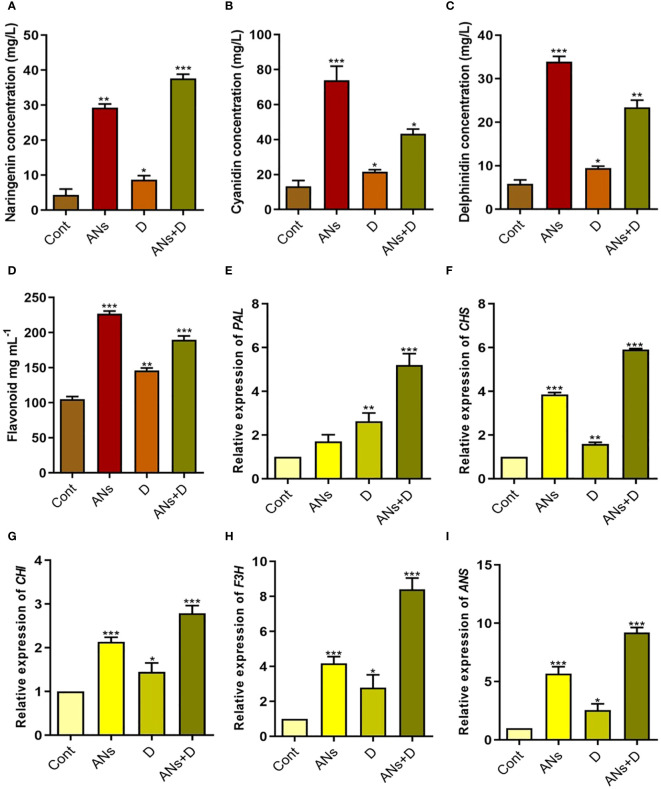
Anthocyanin application induces flavonoid biosynthesis pathway under drought stress. **(A-D)** shows naringenin, cyaniding, delphinidin, and total flavonoids contents respectively, in rice plants. **(E-I)** shows expression level of *PAL*, *CHS*, *CHI*, *F3H*, and *ANS* respectively. Data were analyzed in three independent biological replicates ( ± standard deviation, SD), and the means were compared using Bonferroni Post-Hoc test. * indicates p< 0.05, ** indicates p< 0.01, and *** indicates p< 0.001.

Anthocyanin and naringenin, vital secondary metabolites with ROS-scavenging capabilities, play a pivotal role in stress alleviation. Our investigation unveiled a noteworthy elevation in naringenin, cyanidin, delphinidin, and total flavonoid levels in plants supplemented with anthocyanin, both under normal and drought stress conditions (**Figures 3A–D**). Likewise, non-treated plants also experienced a significant increase in naringenin, cyanidin, delphinidin, and total flavonoids during drought stress compared to control plants. Comparison between anthocyanin-treated and non-treated plants under drought stress revealed a 77% increase in naringenin, 99% in cyanidin, 147% in delphinidin, and 44% in total flavonoids. These results highlight the capability of exogenously applied anthocyanin to enhance the accumulation of these metabolites in response to drought stress.

This observed trend extended to the expression patterns of genes associated with flavonoid biosynthesis (**Figures 3E–I**). Expression levels of *PAL*, *CHS*, *CHI*, *F3H*, and *ANS* were significantly elevated in anthocyanin-treated (ANs), drought-stressed (D), and anthocyanin-treated + drought-stressed (ANs+D) plants compared to control plants, with the exception of *PAL*, which exhibited non-significant upregulation. Particularly under drought stress, expression levels increased by 97% for *PAL*, 270% for *CHS*, 92% for *CHI*, 202% for *F3H*, and 261% for *ANS* in anthocyanin-treated plants relative to non-treated plants. These findings highlight the role of exogenously applied anthocyanin in augmenting the regulation of *PAL*, *CHS*, *CHI*, *F3H*, and *ANS* genes, thereby contributing to the mitigation of drought-induced stress.

### Anthocyanin regulates melatonin, proline, ABA, and SA under drought stress

**Figure 4:**
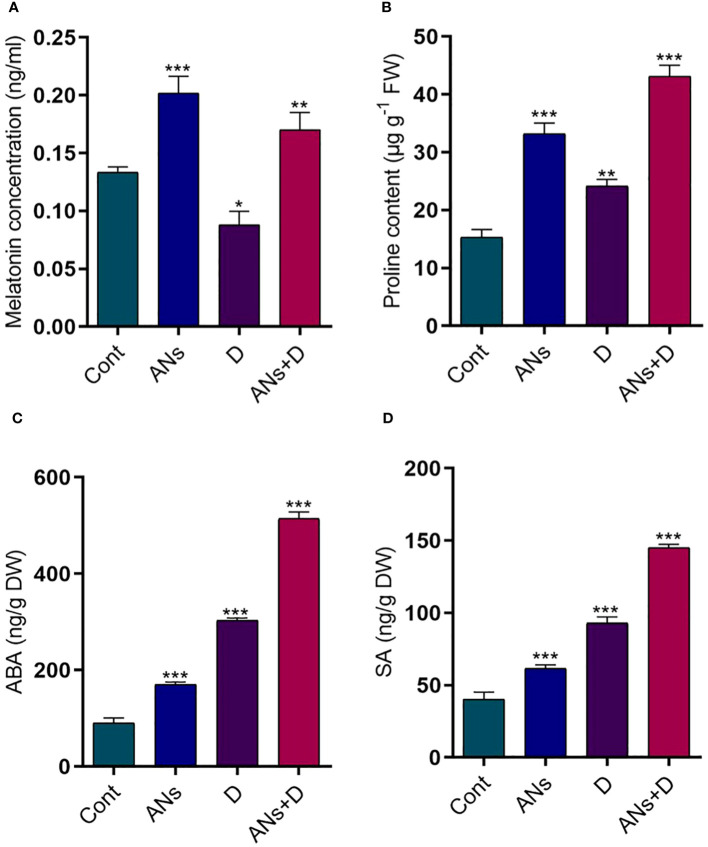
Anthocyanin regulates phyto-hormones and proline content in rice plant during drought stress. **(A)** melatonin, **(B)** proline, **(C)** ABA, and **(D)** SA contents. Data were analyzed in three independent biological replicates ( ± standard deviation, SD), and the means were compared using Bonferroni Post-Hoc test. * indicates p< 0.05, ** indicates p< 0.01, and *** indicates p< 0.001.

Anthocyanins, a crucial class of flavonoids capable with ROS-scavenging properties, play a pivotal role in enhancing plant stress tolerance (Jan et al., 2023a). The current study revealed a remarkable elevation in melatonin levels in both anthocyanin-treated normal and stressed plants. Conversely, a significant reduction in melatonin was observed in non-treated plants exposed to drought stress as compared to control plants (**Figure 4A**). Specifically, melatonin displayed a remarkable 93% increase in ANs+D plants when compared to non-treated plants facing drought stress. Proline contents exhibited a substantial increase in both anthocyanin-treated and non-treated plants under both drought stress and normal conditions, relative to control plants (**Figure 4B**). Anthocyanin treatment was associated with a substantial 78% increase in proline contents compared to non-treated plants during drought stress.

Furthermore, we delved into the impact of drought stress on ABA and SA levels in both treated and non-treated plants, comparing them to control plants (**Figures 4C, D**). Both the ABA and SA were significantly increased in treated and non-treated plants when exposed to drought stress, compared to control plants. While anthocyanin treated plants significantly increased ABA 69% and SA 57% compared to non-treated plants under drought stress condition. In conclusion, the findings highlight the beneficial role of anthocyanins in modulating melatonin levels and proline accumulation, contributing to enhanced stress resilience in plants. Additionally, the observed reductions in ABA and SA levels following anthocyanin treatment suggest a potential regulatory role of anthocyanins in mitigating the impact of drought stress on these signaling molecules, further elucidating the complex interplay between flavonoids and plant stress responses.

### Anthocyanin regulate enzymatic activity of antioxidants and drought responsive genes

**Figure 5:**
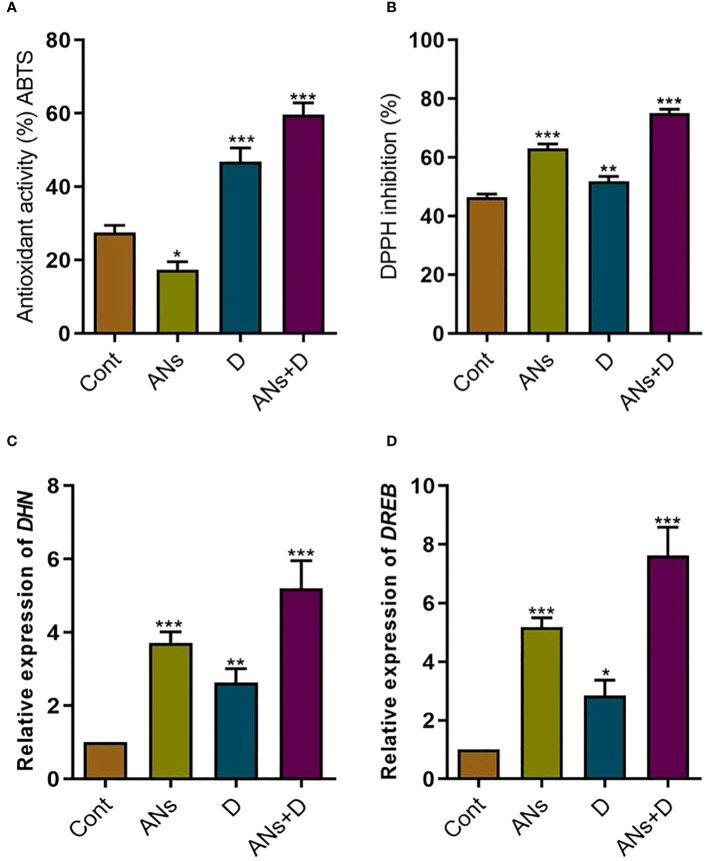
Anthocyanin application significantly regulates antioxidant activity and drought related gene in rice plant. **(A)** ABTS antioxidant capacity, **(B)** DPPH free radical scavenging capacity, **(C)** *DHN* gene expression, and **(D)** *DREB* gene expression. Data were analyzed in three independent biological replicates ( ± standard deviation, SD), and the means were compared using Bonferroni Post-Hoc test. * indicates p< 0.05, ** indicates p< 0.01, and *** indicates p< 0.001.

To gain a clearer understanding of the mechanism underlying anthocyanin-induced mitigation of drought stress in rice plants, we investigated the enzymatic activity of ABTS and DPPH, as well as the expression levels of drought stress-responsive genes such as; *Dehydrin* (*DHN*) and *Dehydration-responsive element-binding protein* (*DREB*) (**Figure 5**). Both treated and non-treated plants exhibited heightened ABTS and DPPH activity under drought stress compared to control plants (**Figures 5A, B**). Furthermore, treated plants demonstrated a 27% increase in ABTS and a 44% increase in DPPH activity under drought stress when compared to non-treated plants. However, anthocyanin significantly reduced ABTS activity and increased DPPH activity compared to control plants under normal conditions. This shows that in normal condition anthocyanin activate only DPPH instead of both the enzymes. Similarly, the expression of *DHN* and *DREB* genes was significantly elevated in both treated and non-treated plants under both normal and drought stress conditions compared to control plants (**Figures 5C, D**). Notably, anthocyanin treatment resulted in a substantial 97% increase in *DHN* and a remarkable 168% increase in *DREB* expression when compared to non-treated plants under drought stress. These finding demonstrates that enhancements in ABTS and DPPH activities, coupled with the upregulation of *DHN* and *DREB* genes, suggest that anthocyanins contribute to the antioxidant defense and stress-responsive gene expression in rice plants under both normal and drought stress conditions. The specific modulation of these responses by anthocyanins highlights their potential as key players in conferring resilience to drought stress in rice.

### Anthocyanin promotes macronutrient contents in rice plant under drought stress

**Figure 6:**
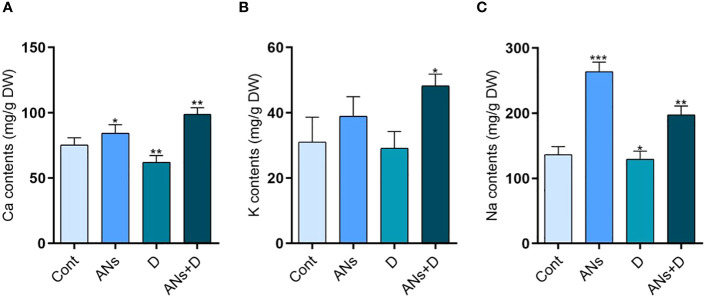
Anthocyanin enhances the accumulation of macronutrients during drought stress in rice plant. **(A-C)** are Ca, K, and Na concentration respectively. Data were analyzed in three independent biological replicates ± SD, and means were compared using Bonferroni Post-Hoc test.*P<0.05,**P<0.001, ***P<0.001.

Na^+^, K^+^, and Ca^+^ represent pivotal macronutrients essential for cell wall and membrane stabilization (Jan et al., 2023b). Our investigation revealed a significant elevation in the levels of all three macronutrients in anthocyanin-treated plants compared to control plants (**Figure 6**). Drought stress imposed a notable reduction of 17% in Ca^+^ and 5% in Na^+^ in non-treated plants subjected to drought stress when compared to control plants. Interestingly, anthocyanin treatment exhibited a remarkable augmentation, enhancing Ca^+^ levels by 59%, K^+^ by 65%, and Na^+^ by 52% compared to non-treated plants under conditions of drought stress. These findings highlight the pivotal role of anthocyanin in boosting drought stress tolerance through the intricate regulation of these crucial macronutrients. The observed enhancements in Na^+^, K^+^, and Ca^+^ concentrations emphasize the potential of anthocyanin as a regulatory factor mitigating the adverse impacts of drought stress on these essential elements. These results provides valuable insights into the mechanistic aspect of anthocyanin-mediated enhancement of drought stress tolerance, shedding light on its potential applications in agricultural practices.

## Discussion

The data presented herein provides insight into a potential mechanism whereby anthocyanin orchestrates the modulation of plant tolerance in response to drought stress. Our investigation reveals that anthocyanin exerts regulatory influence across multiple stress tolerance pathways. Specifically, it impacts antioxidant enzyme activity, initiates the phenylalanine ammonia-lyase (PAL) pathway, influences the accumulation of phytohormones, facilitates melatonin accumulation, modulates drought-related gene expression, and governs the regulation of essential macronutrients. These findings underscore the multifaceted role of anthocyanin in enhancing plant resilience under conditions of drought-induced stress.

Our study demonstrates that the application of exogenous anthocyanin positively influences various key indicators of plant growth under drought conditions. Notable improvements were observed in parameters such as shoot and root length, leaf area, chlorophyll content, relative water content, panicle length and number, as well as seed number and weight (**Figure 1**). These findings suggest that anthocyanin supplementation effectively mitigates the adverse effects of drought stress, fostering normal plant growth and development. Furthermore, our investigation reveals a significant reduction in H_2_O_2_, O_2_^•−^ and MDA contents in drought-stressed plants treated with exogenous anthocyanin, as depicted in **Figure 2**. This reduction underscores the role of anthocyanin in ameliorating oxidative stress associated with drought conditions. The observed decline in ROS and MDA levels is indicative of anthocyanin’s capacity to serve as a reactive oxygen species scavenger, thereby shielding plants from oxidative damage and augmenting their resilience. This aligns with existing literature highlighting the antioxidative properties of anthocyanins, emphasizing their potential to enhance plant sustainability in challenging environmental conditions (Dabravolski and Isayenkov, 2023).

We conducted a comprehensive analysis, monitoring the accumulation of naringenin, cyanidin, delphinidin, total flavonoids, and key pathway genes (*PAL*, *CHS*, *CHI*, *F3H*, and *ANS*) in both anthocyanin-treated and non-treated plants subjected to drought stress. Our findings reveal a consistent upregulation of all examined metabolites and their associated biosynthesis genes in anthocyanin-treated plants compared to their non-treated counterparts under drought stress conditions (**Figure 3**). These results provide evidence that the exogenous application of anthocyanin positively influences the PAL pathway genes, thereby augmenting the endogenous accumulation of anthocyanins. A recent study have reported that, under drought conditions, plants activate survival mechanisms, particularly biosynthesis of secondary metabolites such as phenolics and flavonoids, influencing the accumulation of polyphenolic substances, including anthocyanins and other flavonoids (Park et al., 2023). Our investigation aligns with prior studies demonstrating a significant elevation of anthocyanin levels in pea and grape berry plants under drought stress conditions (Nogués et al., 1998; Castellarin et al., 2007). The stress-induced anthocyanins function as potent free radical scavengers, thereby mitigating oxidative stress associated with drought by facilitating their transport to the vacuole (Zahedi et al., 2021). The proposed role of anthocyanins as osmoregulators in stressed plants involves the regulation of water homeostasis, contributing to enhanced resistance to drought stress. Cirillo et al. (2021) suggested that plant species with higher anthocyanin levels tend to exhibit greater resistance to drought conditions (Cirillo et al., 2021).

Furthermore, our investigation delves into the gene expression of the phenylalanine ammonia lyase (PAL) pathway under drought stress. Our results indicate a significant upregulation of *PAL*, *CHS*, *CHI*, *F3H*, and *ANS* genes in response to exogenous anthocyanin during drought stress. Notably, these genes also exhibited significant enhancement under drought stress in normal conditions (non-treated plants) compared to control plants. This finding is consistent with previous studies, such as the observed increase in flavonoids and relevant genes, including *PAL*, *CHS*, *CHI*, *F3H*, *FLS*, *DFR*, and *ANS*, in Achillea pachycephala Rech.f during drought stress exposure (Gharibi et al., 2019). Similarly, significant induction of flavonoid biosynthesis genes was reported in buckwheat under drought stress (Hou et al., 2019). In accordance with our findings and previous research, we conclude that the heightened levels of anthocyanins and total flavonoids under drought stress are closely associated with the upregulation of flavonoid biosynthesis genes, including *PAL*, *CHS*, *CHI*, *F3H*, *FLS*, *DFR*, and *ANS* (Ma et al., 2014). This suggests a coordinated regulatory mechanism governing the enhanced synthesis of these secondary metabolites in response to drought-induced stress. Further, upon closer examination, it was observed that the expression of drought-responsive genes, namely *DHN* and *DREB*, was notably upregulated with the application of anthocyanin under both normal and drought conditions (**Figures 5C, D**). These findings suggest a positive correlation between *DHN* and *DREB* genes and anthocyanin, indicating a potential involvement of anthocyanin in the regulation of these genes. Even though, regulation of *DREB* on anthocyanin biosynthesis is completely unknown, however a recent study predicted that *DREB* gene expression in *Ammopiptanthus mongolicus* enhanced the accumulation of anthocyanin under drought stress.

The production of phytohormones, notably ABA and SA, is a well-established defense mechanism against drought stress. In our investigation of the relationship between anthocyanin and ABA and SA biosynthesis during drought stress, we observed that the exogenous application of anthocyanin significantly heightened the accumulation of ABA and SA compared to both control and drought-exposed plants (**Figures 4C, D**). Numerous studies have suggested that the drought stress-induced increase in ABA, proline, and sugars may play pivotal roles in anthocyanin biosynthesis (Das et al., 2012; Sperdouli and Moustakas, 2012; González-Villagra et al., 2019). Previous research, such as that on *Torenia fournieri*, has proposed that ABA precedes anthocyanin biosynthesis during drought stress, with ABA induction occurring before the regulation of anthocyanin (González-Villagra et al., 2019). These findings elucidate that ABA induction occurs during periods of drought stress, subsequently triggering the biosynthesis of anthocyanin. However, in our study, the exogenous application of anthocyanin resulted in an enhanced accumulation of ABA in drought-stressed plants, indicating a synergistic association between ABA and anthocyanin during drought stress. While SA is traditionally known for its role in biotic stress, recent studies have implicated its active participation in plant tolerance to drought stress (Brito et al., 2018; Khan et al., 2022). Consistent with these findings, we observed an increase in SA levels in drought-stressed plants compared to control plants. Moreover, anthocyanin-treated plants exhibited even higher SA accumulation under drought stress, suggesting that anthocyanin application enhances SA accumulation in rice during drought stress. In support with previous study, Our study posits that SA may mitigate drought stress by triggering an antioxidant system that alleviates oxidative stress, as reported in *Aristotelia chilensis* plants (González-Villagra et al., 2022).

Interestingly, our results revealed a substantial induction of proline in anthocyanin-treated plants compared to non-treated plants under drought stress (**Figure 4B**). Proline, an amino acid with a well-established response to osmotic stress in plants, acts as a crucial component in stress reduction (Cirillo et al., 2021). This includes functioning as a buffer for cellular redox potential, serving as a ROS scavenger, stabilizing proteins and membranes, and inducing genes related to drought and salt stress (Satoh et al., 2002; Carillo, 2018). Additionally, proline can be rapidly metabolized when no longer needed, providing essential resources for the recovery and repair of stress-induced damages (Carillo et al., 2008). Our findings, aligned with previous reports, underscore the association between increased anthocyanin accumulation and a notable metabolic shift involving proline in response to drought stress.

Melatonin, classified as an indole molecule, exhibits pronounced free radical scavenging capabilities, positioning it as a pivotal candidate for mitigating oxidative stress (Sun et al., 2023). Analogous to proline, melatonin functions as an inhibitor of oxidative stress by scavenging ROS induced by drought stress (Sharma and Zheng, 2019). The outcomes of our investigation reveal that the application of anthocyanin leads to an augmentation of melatonin levels in rice plants subjected to drought stress, as depicted in **Figure 4A**. Intriguingly, a recent study underscores the reciprocal relationship between melatonin and anthocyanin. It elucidates that melatonin not only induces the expression of anthocyanin biosynthesis genes, fostering the accumulation of anthocyanin, but also manifests stress-reducing properties through chelation mechanisms (Sun et al., 2023). The intricate interplay observed between melatonin and anthocyanin suggests a synergistic dynamic that may contribute significantly to enhancing overall stress tolerance in plants, particularly under drought conditions. Numerous researchers have posited diverse mechanisms through which melatonin imparts drought stress tolerance. These mechanisms encompass the preservation of water, maintenance of cell membrane integrity, reduction of cytotoxic biochemicals such as H_2_O_2_ and MDA, and augmentation of antioxidant enzyme activity (Yan et al., 2016; Wei et al., 2018; Khan et al., 2020). Collectively, these actions underscore melatonin’s multifaceted role as a protective agent during periods of drought-induced stress. In alignment with these findings, a contemporary study anticipates that the application of anthocyanin serves as a catalyst for drought stress tolerance by facilitating the accumulation of melatonin. This predictive insight highlights the potential synergistic relationship between anthocyanin and melatonin, offering a promising avenue for bolstering plant resilience in the face of challenging environmental conditions.

Plants actively accumulate essential macronutrients such as Na^+^, K^+^, and Ca^+^ within the cytosol to support fundamental physiological processes. While high levels of K^+^ initially facilitate increased water absorption during drought stress, a subsequent reduction in K^+^ levels is observed in the later stages (Fayyaz et al., 2013). Ca^+^ plays a pivotal role in plant adaptation to adverse conditions under water-deficit circumstances, serving as a regulatory element in plant cell metabolism (Bowler and Fluhr, 2000). The application of Ca^+^ has been shown to enhance drought resistance by inhibiting the synthesis of active oxides, preserving the integrity of the plasma membrane, sustaining normal photosynthesis, and modulating the metabolism of various plant hormones and other essential chemicals (Pathak et al., 2020). Some studies have indicated that elevated Na^+^concentrations can impede the accumulation of K^+^, elevate osmotic pressure, induce oxidative damage, and hinder productivity, growth, and metabolic activities (James et al., 2011; Jan et al., 2021b). Contrary to the typical response observed under drought stress, our findings reveal a simultaneous reduction in these macronutrients in untreated plants, whereas anthocyanin-treated plants exhibit a significant increase in Ca^+^, K^+^, and Na^+^ levels concurrently (**Figure 6**). The supplementation of anthocyanins appears to mitigate the impact of drought stress by regulating the levels of these essential macronutrients in rice plants. However, a comprehensive understanding of the molecular mechanisms underlying this regulatory effect requires further investigation.

## Conclusion

In conclusion, our study has shown that the application of anthocyanins significantly enhances the phenotypes of rice plants, mitigates the effects of drought-induced oxidative stress, and modulates crucial physiological and molecular responses under drought conditions. Anthocyanin-treated plants exhibit notable improvements in various morphological and biochemical traits, including increased shoot and root length, chlorophyll content, relative water content, panicle number, and seed weight. Additionally, anthocyanin application reduces oxidative damage by lowering levels of reactive oxygen species, lipid peroxidation, and enhancing antioxidant enzyme activities. The observed upregulation of anthocyanins, flavonoids, and associated biosynthetic genes points to their role in alleviating stress, while changes in melatonin, proline, ABA, and SA levels highlight their regulatory effects on stress-responsive pathways. Furthermore, anthocyanin positively influences the enzymatic activity of antioxidants and the expression of drought-responsive genes. Importantly, anthocyanin treatment promotes the accumulation of essential macronutrients, such as Na^+^, K^+^, and Ca^+^, enhancing plant resilience to drought stress. These findings collectively highlight the multifaceted role of anthocyanins in conferring drought stress tolerance in rice plants, providing valuable insights for potential applications in sustainable agricultural practices.

## Data availability statement

The raw data supporting the conclusions of this article will be made available by the authors, without undue reservation.

## Author contributions

SalA: Formal analysis, Software, Writing – review & editing. RJ: Methodology, Software, Validation, Writing – original draft, Writing – review & editing. SajA: Validation, Visualization, Writing – review & editing. ZK: Formal analysis, Software, Writing – review & editing. KK: Conceptualization, Funding acquisition, Resources, Supervision, Writing – review & editing. L: Conceptualization, Software, Validation, Writing – review & editing.
